# Grandzüge Der Pathologischen Histologie

**Published:** 1855-03

**Authors:** 


### BIBLIOGRAPHICAL NOTICES.

*Grandzüge der Pathologischen Histologie.* Von Dr. CARL WEDL. Wien: 1854.

*Outlines of Pathological Histology.* By DR. CHARLES WEDL. Vienna: 1854.*

Pathological Anatomy has of late years received much valuable aid from microscopical and chemical research; indeed, the coarser study of the pathological anatomy of the organs has been rapidly yielding to the minute study of the pathological tissues, or Pathological Histology. Yet, notwithstanding the fact that numerous observers are engaged in the study of this new and interesting branch of science, we have up to the present day not been in possession of a systematic treatise on the subject, as all observations are recorded in separate monographs, or are found scattered throughout the treatises on General Pathology. Dr. Wedl, the author of the volume now before us, determined, therefore, to write a work indicating the "Outlines" of Pathological Histology, and to base it mainly on personal observation; a task which enormous industry and vast opportunity alone could have brought to anything like a satisfactory termination. Of the possession of the former, the work itself is the best evidence, the latter was supplied by the extensive dead-house at Vienna, which has been rendered so famous through the labors of Rokitansky, and to which Dr. Wedl has been for years attached.

* We are bound in courtesy towards the author to state that the review of this work, a copy of which he politely forwarded to us, has been by accidental circumstances prevented from appearing in print at an earlier period.—REVIEWER.

The work itself is a large octavo, divided into two parts: one discussing General, the other Special Pathological Histology. The first part extends over 108 pages, and is taken up with a consideration of the general pathological conditions of the circulation, and with the formation, in abnormal developments, of cells, fibres, areolar tissue, papillary excrescences, vessels and cysts. The author's views are here well and clearly stated; this whole part, indeed, besides containing many valuable original observations, may be considered as an admirable *resumé* of the present state of general pathological histology. As not the least important chapter in it, we regard the excellent introduction, comprising an account of the means and the methods to be employed in the investigation of pathological tissues. Yet we cannot help stating this part of Dr. Wedl's work to be inferior in value as an original production to the second division, or the one treating of Special Pathological Histology. This is again subdivided into six groups. In the first, or "Inorganic Formations," the different crystals and concretions met with in the human fluids are described. The second group includes the "Atrophies" of tissue and of the blood; the third, the "Hypertrophies;" the fourth, the "Exudations" and their products; the fifth, the "New Formations," as pus, tubercle, bone, etc., whilst the sixth group is devoted to a description of the "Vegetable and Animal Parasites."

Having thus indicated the general contents of the volume before us, let us now examine more closely some of its more important parts. The basis of Pathological Histology, after all, must be the study of the cells, and the ultimate end which pathologists must strive to reach, will be to determine the changes which imperfect nutrition, and other causes, produce on the cell-elements and the tissues they form. Let us, for example, take up the most frequent of the "Atrophies" occurring in cells or in any formed tissue, and dependent upon faulty nutrition, viz., *fatty degeneration*. This in cells consists of the appearance within the walls of a quantity of a fine molecular fat, or sometimes even of distinct oil-globules. This deposit results in an atrophy of the cell, and is generally supposed to affect primarily the nucleus, which it causes to shrivel up and disappear. This primary atrophy of the nucleus Wedl denies, and every microscopist must agree with him at least in this particular, that it is not uncommon to meet with whole groups of cells nearly over-distended by fatty infiltration, in which the nuclei are as distinct as in normal cells, and perfectly unaltered in shape. In determining this point, it is of the greatest importance to bear in mind that the cells in many organs contain normally much oil. Fatty degenerations of tissues occurs most frequently in muscular fibre and in the blood-vessels. The fatty infiltration and consequent softening of the coats of the capillaries of the brain is probably a not unfrequent cause of apoplexy.

The formation of *vessels* in pathological tissues Wedl describes as occurring free in an exudation, without any necessary connection existing with previously formed capillaries. The first step, he thinks, is the formation in this exudation of branched cells, whose processes then becoming connected, the walls are absorbed, and thus a communicating system of primary capillaries is developed, from which then again others may arise. If this view be correct, the process of formation of vessels in pathological tissues, would correspond closely to the development of vessels which Schwann and Kölliker describe as occurring normally in the tail of a tadpole; it would indeed be but a slight modification of the old view of John Hunter on the subject. The diameter of these newly-formed vessels is generally greater than that in those of normal tissue; in structure they consist of a layer of fibre-cells with oblong, longitudinally arranged nuclei. In the arachnoid, this simple structure of the vessels is, remarkably well shown, as here they are more like capillaries, and never possess either a longitudinal or annular fibrous coat.

A subject which Wedl has evidently carefully investigated is the formation of *cysts*. The word cysts he restricts to an

"excessive augmentation of the spaces in areolar tissue with a papillary new formation. Hence cysts are more readily seen in organs in which new formations of fibrous tissue and of papillary excrescences frequently occur, as in the thyroid gland, mammary gland, ovary, kidney, etc. Yet this does not exclude their formation in any organ, only they will be more rarely seen in parts, in which new formations of areolar tissue and papillary excrescences seldom occur. We regard, therefore, cysts never as primary, but always as secondary formations." (p. 102.)

Now although this view of the nature of cysts may be convenient to explain their formations, we cannot regard it as altogether based upon strict pathological research. Primary cysts, or as

Paget* terms them, "autogenous cysts," occur in parts where certainly at first no fibrous tissue existed; thus we see cysts originating in soft cancerous tumors, evidently from the transformation of the morbid structure, and not from the expansion of the areolar spaces of the fibro-cellular tissue. In their minute structure, cysts consist of an external coat of fibrous tissue, lined by a layer of cells like those of pavement epithelium. This epithelial lining is, according to Rokitansky†, frequently absent in large cysts. Wedl describes it to be invariably wanting where parts of the fibrous coat project into the cyst-cavity. Seated on these projections are sometimes "dendritic" formations, which Wedl has observed to acquire sometimes so large a size as nearly to fill the cavity of the cyst, and by their transformation into smaller cysts, (in a manner presently to be described,) to give rise to compound cystic growths. In the external coat, different organs, as hair, teeth, etc., may form, which, by subsequently projecting into the cyst, cause the singular phenomenon so frequently seen of cystic growths containing, as it were, these structures.

As we have already stated our author believes cysts to be invariably secondary formations. He conceives first an exudation to occur from the blood-vessels into the areolæ of the fibro-areolar tissue. This exudation is then transformed into fibre-cells and vessels, whilst a fresh exudation completely fills up the space in the areolar tissue. The fibre-cells in the walls of this former space are now further developed into papillary excrescences with dendritic vegetation, whilst at the same time the effused blastema forms epithelial cells, which serve as a lining to the minute cyst. This process may be repeated in several areolæ, and a compound cyst results, whilst if the exudation have occurred into a large areola, which encloses smaller, the effused fluid will cause an absorption of their fibrous walls giving rise to one large cyst with irregular projections of the fibrous coat. These observations and the theory based upon them, we find very difficult to reconcile with the theory on cystic formations recently advanced by Rokitansky, (loc. cit.) who, as the result of much careful investigation, declares all cysts to be primitive

* Lectures on Surgical Pathology, Phil. 1854.

† Ueber die Cyste. Vienna, 1852.

formations, and traces their origin from a nucleus and a structureless vesicle, containing a clear fluid, upwards into a perfectly formed growth.

Amongst the group of "exudations" we find *colloid* mentioned. This colloid, which has again quite recently, through Virchow's discovery, become a substance of such disputed import, is a transparent, apparently homogenous exudation, resembling gelatin, and is mainly found in cysts, especially those of the thyroid gland. Colloid, when it coagulates, presents under the microscope, the appearance of large bodies, consisting of concentric layers of an opaque substance, described by most authors as "concentric colloid-corpuscles;" by Kölliker and Virchow under the name of "corpora amylacea." The distinguishing micro-chemical reaction of these corpuscles is, that they are not acted upon by acetic acid nor diluted alkaline solution, whilst strong alkalies generally dissolve them. Wedl has met with this exudation in many tissues and fluids. He distinguished round colloid-corpuscles, distinctly granular, and possessed, towards the circumference, of radiating striæ, in cysts of an atrophied kidney, also (although in this case they were more hyaline and oval) in an inflamed choroid coat after the removal of the pigment. As belonging to colloid he also enumerates those peculiar reddish, concentric bodies which Hassall has depicted as existing in the prostate gland of aged people, but which, long ago, Virchow proved to be a proteinaceous substance, analogous to that found in the seminal fluid. He further classes as colloid the corpuscles found in the peculiar pathological condition known as "waxy spleen," and also the corpora amylacea of the brain.

It will be seen from this but partial enumeration of the corpuscles which Wedl has grouped as colloid, that he considers this exudation to be unusually prevalent in pathological productions. We doubt not, however, that many substances which are thus considered colloid, are, in reality, concretions of different substances. We have already indicated, that the corpuscles in the prostate are proteinaceous, the recent discoveries of Virchow* permit us further to state that the corpora amylacea of

* See Virchow's Archiv., Vol. vi., 1, 2, 3; and Medical Examiner, 1854, Vol. x. pp. 267 and 333.

the brain and the corpuscle in waxy spleen do not belong to the colloid group, but are in reality substances like vegetable cellulose, for on addition of iodine and sulphuric acid they assume the peculiar characteristic violet color of this substance. That all the "colloid" corpuscles met with in other situations are, on the other hand, not cellulose, is nearly settled. Virchow tested by iodine and sulphuric acid the concentric bodies in the concretions of the prostate and of the vesiculæ seminales, and they remained unchanged, whilst again Busk* and Donders,† who repeated Virchow's observations on the amylaceous brain-matter consider his "cellulose corpuscles" to be starch granules, and Meckel‡ affirms them to be cholesterine. Now from this chaos of conflicting opinions to which Virchow's discovery has given rise, we must look to chemistry to extricate us, yet we are already justified in concluding that the corpuscles which have hitherto been described as colloid, are in reality masses or crystals of many different substances, including albuminoid masses, starch-granules, and cellulose.

The subjects we have discussed are for the most part treated of in the first division of Dr. Wedl's work ; the second part, whose contents we have already indicated, is replete with many valuable original observations, but our space compels us, instead of following the author throughout in his descriptions, to consider merely one of the most important of his groups, the "New Formations." These Wedl has classed under ten heads ; first, the granular cells and masses ; the second, pus ; the third, tubercle ; the fourth, new formations in typhoid fever ; the fifth, new formations of fibrous tissue, as on the skin and in the different organs ; the sixth, new formations of fatty tissue ; the seventh, cholesteatoma ; the eighth, osseous and cartilaginous new formations ; the ninth, formation of tooth substances ; and lastly, the tenth, cancer. Now amongst these, again, the formations which, on account of their frequent occurrence and importance, recommend themselves more particularly to the attention of the pathologist, are pus, tubercle, typhoid fever products and cancer.

* Microscopical Journal, 6 Jan., 1854, p. 101.
† Nederlandsch Lancet, 1854.
‡ Berliner Annalen, 1854.

*Pus*, Wedl regards, and he agrees with Vogel, Paget and nearly all modern observers in this particular, to be a true degeneration of an inflamatory plasma, and not simply, as frequently supposed, an increase of white corpuscles of the blood. Mucus, as it is met with in pathological products is but modified pus. It contains more mucin in its composition, but the corpuscles are identical, since acetic acid will render nuclei apparent in both.

Formerly all small elevations occurring in any tissue were denominated *tubercle.* Now we design as tubercle, masses analogous to those found in the lung, which are clinically connected with a certain set of well-marked symptoms. The form tubercles present is very various. Some are small grey elevations, others distinctly isolated yellow masses, some are really infiltrations into tissues, others are merely loosely surrounded by the parts in which they are deposited. Yet all these varieties agree in this particular, that they never contain vessels, and that they consist microscopically of granules, a firm proteinaceous blastema in flakes, of nuclei and imperfectly formed granular bodies and cells. The difference in their external appearance led Rokitansky to consider grey and yellow tubercles as two distinct varieties of fibrinous lymph, and induced Robin to advocate the singular view that tubercles are never grey, but that true tubercular matter is always yellow. The difficulty of distinguishing between tubercles and some forms of degenerate lymph, and arrested cell formation is sometimes very great; and, in consequence, many pathologists have sought for special corpuscles by which tubercle might be recognised. Thus it was that Lebert gave the name of "tubercle corpuscle" to the peculiar irregular granular bodies without distinct nuclei and uninfluenced by acetic acid, so frequently met with in tubercular masses. Wedl does not consider these bodies of much diagnostic importance, for he affirms that he has met with the same bodies in the products found in the intestines in typhoid fever, and regards them only as cells arrested in their development. He considers, indeed, the microscopical diagnosis of tubercle only possible by a system of microscopical exclusion.

*See Bernard and Robin on the Blood. Phila. 1854.

"Positive, characteristic elements tubercle does not possess. The microscopical diagnosis must therefore be made by exclusion. If we meet with a pathological new-formation consisting only of the above named constituents and fully formed cells of various shapes, if blood vessels or blood spaces, or a fibrous stroma enveloping the cells be absent, we are sure that it is a pure tuberculous formation we have before us. If, on the other hand, the imperfecly formed elements are only found in part of a new formation, for example, of a cancer, while perfectly developed cells of different shapes, along with blood vessels and areolar tissue are seen in other portions, we would regard this formation as a cancer, in part of which the organization had not progressed very far, and had remained, as it were, stationary, in the same low grade as it does in the formation of tubercle." (p. 367.)

When tubercle has existed for any length of time, its general tendency is to soften, or else it changes into a calcareous substance. Softened tubercle resembles pus, but does not contain the same corpuscles as the latter; for, examined microscopically, it consists of molecules, oil globules, free nuclei and imperfectly developed cells. In calcified tubercle, Wedl has found, besides the calcareous salts deposited in the centre of the hardened mass, oil globules, brownish pigment and crystals of cholesterine.

As closely connected with tubercle, both in regard to its mode of development and structure, Wedl describes the *deposits* that take place in *typhoid* fever in Peyer's glands, in the mesenteric glands, and between the mucous and muscular tissue of the intestine. The elements he has observed in these infiltrations are cells 0.008 to 0.024 mm. in size, of a roundish form, and containing one or sometimes several oval nuclei. These cells also enclose many fine granules, sometimes, also, oil globules, which conceal the nucleus and fill up the whole of the interior of the cell. Besides these roundish cells, which are the most abundant, he finds in typhoid fever infiltrations, many spindle-shaped cells with large oval nuclei and nucleoli. In many cases, instead of masses of fully developed cells, Wedl states that he has met with granular bodies, similar to the ones Lebert has described as "specific" tubercle-corpuscles. On the surface of the ulceration, the organic new-formation is not distinct. Here nuclei of varying size and a few oval cells are found imbedded in a molecular mass containing also fat-globules.

The last of the new-formations which Wedl describes is *cancer*. We cannot follow our author in his elaborate description of the various forms of cancer met with in the human economy, but will merely state, that he is one of those who deny the specificity of the cancer cell. The microscopical diagnostic marks of cancer on which Wedl seems to lay most stress, are the study of the evolution or involution of the cells, or in other words, their vital phenomena, their growth, their metamorphosis, their multiplication, and the formation of other tissues in the morbid structure. Without entering here into the much vexed question of the cancer cell, we would yet, in this particular, venture to suggest to the author, the employment in his investigations of higher magnifying powers than he seems to have used.

If in conclusion we were called upon to state our impression of the general merits of Prof. Wedl's work, we must express the sincere pleasure we derived from its perusal. The work is, as far as we are aware, the first that has as yet appeared upon pathological histology, but independently of that, it will always retain a high stand on account of the many original and carefully made observations it contains. The style is plain and distinct, the wood-cuts are the best we have seen anywhere, but in the getting up of the book we miss much a proper index.

